# The performance of EuroSCORE II in CABG patients in relation to sex, age, and surgical risk: a nationwide study in 14,118 patients

**DOI:** 10.1186/s13019-023-02141-4

**Published:** 2023-01-19

**Authors:** Martin Silverborn, Susanne Nielsen, Martin Karlsson

**Affiliations:** 1 Department of Molecular and Clinical Medicine, Institute of Medicine, Sahlgrenska Academy, University of Gothenburg, Gothenburg, Sweden; 2 Department of Cardiothoracic Surgery, Sahlgrenska University Hospital, SE-41345 Gothenburg, Sweden

**Keywords:** Coronary artery bypass grafting, EuroSCORE, Risk stratification, Mortality

## Abstract

**Background:**

To determine the discriminative accuracy and calibration of EuroSCORE II in relation to age, sex, and surgical risk in a large nationwide coronary artery bypass grafting (CABG) cohort.

**Methods:**

All 14,118 patients undergoing isolated CABG in Sweden during 2012–2017 were included. Individual patient data were taken from the SWEDEHEART registry. Patients were divided by age (< 60, 60–69, 70–79, ≥ 80 years), sex, and surgical risk (low: EuroSCORE < 4%, intermediate: 4–8%, high: > 8%). Discriminative accuracy was determined by the area under the receiver operating characteristic curve (AUC) and calibration by the observed/estimated (O/E) mortality ratio at 30 days.

**Results:**

AUC and O/E ratio were 0.82 (95% CI 0.79–0.85) and 0.58 (0.50–0.66) overall, 0.82 (0.79–0.86) and 0.57 (0.48–0.66) in men, and 0.79 (0.73–0.85) and 0.60 (0.47–0.75) in women. Regarding age, discriminative accuracy was highest in patients aged 60–69 years (AUC: 0.86 [0.80–0.93]) but was satisfactory in all groups (AUC: 0.74–0.80). O/E ratio varied from 0.26 for patients > 60 years to 0.90 for patients > 80 years. Regarding surgical risk, AUC and O/E ratio were 0.63 (0.44–0.83) and 0.18 (0.09–0.30) in low-risk patients, 0.60 (0.55–0.66) and 0.57 (0.46–0.68) in intermediate-risk patients, and 0.78 (0.73–0.83) and 0.78 (0.64–0.92) in high-risk patients.

**Conclusions:**

EuroSCORE II had good discriminative accuracy independently of sex and age, but markedly overestimated mortality risk, especially in younger patients. Accuracy and calibration were better in high-risk patients than in low-risk and intermediate-risk patients.

**Supplementary Information:**

The online version contains supplementary material available at 10.1186/s13019-023-02141-4.

## Introduction

Several risk stratification models based on patient characteristics, comorbidities, and type of surgical procedure have been developed to estimate the mortality risk after cardiac surgery [1]. The European System for Cardiac Operative Risk Evaluation (EuroSCORE), first introduced in 1999, was designed to improve patient selection and became widely adopted [2]. However, as perioperative and postoperative care improved, the discriminative accuracy and calibration of EuroSCORE I decreased. A new version, EuroSCORE II, which outperforms EuroSCORE I for risk stratification, was therefore introduced in 2011 [3]. Today, EuroSCORE II and the Society of Thoracic Surgery Predicted Risk of Mortality (STS-PROM) are the most widely recognized and utilized risk stratification tools [4, 5]. EuroSCORE II and STS-PROM have comparable discriminative accuracy and calibration regarding in-hospital and 30-day mortality in coronary artery bypass grafting (CABG) and in aortic valve replacement (AVR) patients [5–8].

Previous analyses of cardiac surgery risk scores, including EuroSCORE II, have noted that the scores overestimate the risk of death after CABG in octogenarians [9–14] and in high-risk patients [15, 16]. However, most of these studies were performed in single-centre cohorts of limited size, and large contemporary population-based studies are lacking. In the present study, we hypothesized on the basis of these previous studies that EuroSCORE II would perform less well in octogenarians and in high-risk patients. To test this hypothesis, we assessed the predictive accuracy and calibration of EuroSCORE II in different age groups, in men and women, and in patients with low, intermediate, and high surgical risk, using a large nationwide cohort of CABG patients.

## Material and methods

### Study population

All consecutive patients > 18 years of age who underwent first-time isolated CABG in Sweden between 1 January 2012 and 30 November 2017 were identified in the Swedish Cardiac Surgery Registry [17], which is part of the Swedish Web System for Enhancement and Development of Evidence-Based Care in Heart Disease Evaluated According to Recommended Therapies registry (SWEDEHEART) [18]. All patients were followed up until death, emigration, or 31 December 2017, whichever occurred first. The study cohort were divided into groups based on age at the time of CABG (< 60, 60–69, 70–79, and ≥ 80 years), sex, and risk group according to the EuroSCORE II surgical risk, with low risk defined as EuroSCORE < 4%, intermediate risk as 4–8%, and high risk as > 8%.

### Data sources

Individual patient data from two nationwide registries were merged on the basis of the personal identification number which all Swedish residents are given at birth or shortly after immigration [19]. Operative details and patient characteristics including EuroSCORE II were extracted from the Swedish Heart Surgery Registry, which prospectively collects detailed information, including risk stratification, on all cardiac surgery patients and operations performed in Sweden since 1992 and has a coverage of 98–99% [17]. Mortality was extracted from the Cause of Death register, which has collected information on date and cause of death based on ICD codes since 1961 [20].

### EuroSCORE II

EuroSCORE II estimates the 30-day mortality risk after cardiac surgery, expressed as a percentage. The variables included in EuroSCORE II are age, sex, presence of renal impairment, extracardiac arteriopathy, poor mobility, previous cardiac surgery, chronic pulmonary disease, active endocarditis, critical preoperative state, insulin-treated diabetes mellitus, New York Heart Association (NYHA) class of heart failure, unstable angina defined as Canadian Cardiology Society (CCS) class 4 angina, left ventricular function (LVEF; > 50%, 30–50%, 20–30%, < 20%), recent myocardial infarction (within 90 days), pulmonary hypertension, urgency of the procedure, weight of the intervention, and surgery on the thoracic aorta [3].

### Outcome

The outcome was all-cause mortality defined as any death occurring between the start of surgery and 30 days after isolated CABG. The expected 30-day mortality, based on the calculated EuroSCORE II for each patient, was compared with the observed 30-day mortality.

### Statistical analysis

Continuous variables were described as means and standard deviations and categorical variables as numbers and percentages. The discriminative accuracy was calculated with c-statistics [21] from a logistic regression and reported as the area under the receiver operating characteristic curve (AUC) with 95% confidence intervals (CIs), both for all patients and stratified by age group, risk group, and sex. Receiver operating characteristic curves and AUC were used to analyse the sensitivity and specificity of expected versus observed mortality within 30 days after surgery. The observed 30-day all-cause mortality was compared with the expected 30-day mortality, based on the calculated EuroSCORE II for each patient. The comparison was achieved by calculating the ratio of observed versus estimated mortality (O/E ratio) for all patients and for the respective groups. In addition, 95% CIs were constructed for the ratios with the bootstrap percentile method using 1000 bootstrapped samples. All tests were two-sided and conducted at the 5% significance level. All statistical analyses were performed using version 9.4 of SAS (Cary, NC).

## Results

### General

The study population consisted of 14,118 consecutive CABG patients. Their mean age was 68.5 years, and 18.3% were women. Baseline characteristics for patients are presented according to age in Table 1, and according to sex and surgical risk in Additional file 1: Tables S1 and S2. The proportions of comorbidities increased by age group except for diabetes, which was less common in the more elderly patients (Table 1). The proportions of men in each EuroSCORE II surgical risk score category were: low risk 43.6%, intermediate risk 49.5%, and high risk 6.8%. The corresponding proportions for women were 19.9%, 63.7%, and 16.4%, respectively (Additional file 1: Table S1). Baseline characteristics by risk group are given in Additional file 1: Table S2.

**Table 1:** Baseline demographics and EuroSCORE II variables among the CABG patients, overall and by age group

	All patients n (%)	< 60 y n (%)	60–69 y n (%)	70–79 y n (%)	≥ 80 y n (%)
No of patients	n = 14,118	n = 2245	n = 4867	n = 5657	n = 1349
Age at surgery, mean (SD)	68.5 (8.9)	53.6 (4.9)	65.1 (2.8)	74.1 (2.8)	82.2 (2.0)
Sex					
Men	11,547 (81.8)	1933 (86.1)	4101 (84.3)	4495 (79.5)	1018 (75.5)
Women	2571 (18.2)	312 (13.9)	766 (15.7)	1162 (20.5)	331 (24.5)
*Medical history*					
Previous heart surgery	176 (1.2)	27 (1.2)	56 (1.2)	86 (1.5)	7 (0.5)
BMI in kg/m^2^, mean (SD)	27.6 (4.2)	28.8 (4.5)	28.0 (4.2)	27.2 (4.0)	26.4 (3.7)
Preoperative dialysis	196 (1.4)	45 (2.0)	69 (1.4)	71 (1.3)	11 (0.8)
Previous PCI	2583 (18.3)	498 (22.2)	936 (19.3)	985 (17.4)	164 (12.2)
Recent myocardial infarction	6711 (47.6)	1078 (48.1)	2183 (44.9)	2719 (48.1)	731 (54.2)
Diabetes	4136 (29.3)	679 (30.3)	1519 (31.2)	1620 (28.6)	318 (23.6)
Hypertension	2896 (77.1)	381 (64.8)	995 (77.7)	1264 (79.8)	256 (83.9)
Atrial fibrillation	689 (6.8)	25 (1.5)	162 (4.7)	366 (8.8)	136 (15.0)
Missing	449 (4.4)	67 (4.1)	163 (4.7)	178 (4.3)	41 (4.5)
Previous stroke	1079 (7.7)	60 (2.7)	300 (6.2)	531 (9.4)	188 (14.0)
Chronic respiratory disease	1165 (8.3)	124 (5.5)	386 (7.9)	539 (9.5)	116 (8.6)
Extracardiac arteriopathy	1103 (7.8)	83 (3.7)	314 (6.5)	559 (9.9)	147 (10.9)
Serum-creatinine, mean (SD)	91.6 (52.9)	89.6 (72.3)	89.6 (54.3)	92.9 (46.1)	96.7 (31.4)
Poor mobility	351 (2.5)	22 (1.0)	107 (2.2)	186 (3.3)	36 (2.7)
Critical preoperative state	299 (2.1)	51 (2.3)	76 (1.6)	137 (2.4)	35 (2.6)
NYHA class					
I	3345 (23.7)	579 (25.8)	1247 (25.6)	1244 (22.0)	275 (20.4)
II	4795 (34.0)	795 (35.5)	1689 (34.7)	1899 (33.6)	412 (30.6)
III	4686 (33.2)	678 (30.3)	1527 (31.4)	1975 (34.9)	506 (37.6)
IV	872 (6.2)	118 (5.3)	265 (5.4)	357 (6.3)	132 (9.8)
CCS class 4 angina	1948 (13.9)	291 (13.0)	636 (13.1)	772 (13.7)	249 (18.6)
Left ventricular function					
Normal	9625 (68.2)	1574 (70.2)	3413 (70.1)	3814 (67.4)	824 (61.2)
31%–50%	3723 (26.4)	546 (24.4)	1187 (24.4)	1551 (27.4)	439 (32.6)
21%–30%	629 (4.5)	93 (4.1)	219 (4.5)	246 (4.3)	71 (5.3)
≤ 20%	134 (0.9)	29 (1.3)	47 (1.0)	45 (0.8)	13 (1.0)
Pulmonary hypertension					
< 30 mmHg	11,256 (93.0)	1847 (95.5)	3843 (93.1)	4535 (92.5)	1031 (90.4)
30–55 mmHg	742 (6.1)	79 (4.1)	251 (6.1)	325 (6.6)	87 (7.6)
> 55 mmHg	104 (0.9)	8 (0.4)	32 (0.8)	42 (0.9)	22 (1.9)
Urgency of the procedure					
Elective	6789 (48.5)	1043 (46.8)	2450 (50.8)	2744 (48.9)	552 (41.4)
Urgent	6572 (47.0)	1056 (47.4)	2199 (45.6)	2613 (46.6)	704 (52.9)
Emergency	558 (4.0)	113 (5.1)	160 (3.3)	222 (4.0)	63 (4.7)
Salvage	74 (0.5)	15 (0.7)	14 (0.3)	32 (0.6)	13 (1.0)
ECC	13,935 (98.7)	2224 (99.1)	4819 (99.0)	5570 (98.5)	1322 (98.0)
EuroSCORE II log					
< 4%	12,412 (87.9)	2147 (95.6)	4584 (94.2)	4786 (84.6)	895 (66.3)
4–8%	1145 (8.1)	58 (2.6)	178 (3.7)	604 (10.7)	305 (22.6)
> 8%	561 (4.0)	40 (1.8)	105 (2.2)	267 (4.7)	149 (11.0)
*Dead within 30 days*					
All patients	205 (1.5)	9 (0.4)	38 (0.8)	102 (1.8)	56 (4.1)
Men	146 (1.3)	5 (0.3)	27 (0.7)	71 (1.6)	43 (4.2)
Women	59 (2.3)	4 (1.3)	11 (1.4)	31 (2.6)	13 (3.8)

Categorical variables presented as n (%), continuous variables presented as mean (SD). *BMI* body mass index; *NYHA class* New York Heart Association class of heart failure; *CCS* Canadian Cardiovascular Society Functional Classification of Angina; *ECC* extra corporal circulation

### Mortality

Overall, the actual 30-day mortality was 1.5% for all patients, 1.3% in men, and 2.3% in women (Table 1). The 30-day mortality increased with age (< 60 years: 0.4%, 60–69 years: 0.8%, 70–79 years: 1.8%, > 80 years: 4.1%; Table 1) and with risk score (low: 0.2%, intermediate: 1.3%, high: 8.0%; Supplementary Table 2).

### Performance of the EuroSCORE II model in CABG patients by age group

**Fig. 1:**
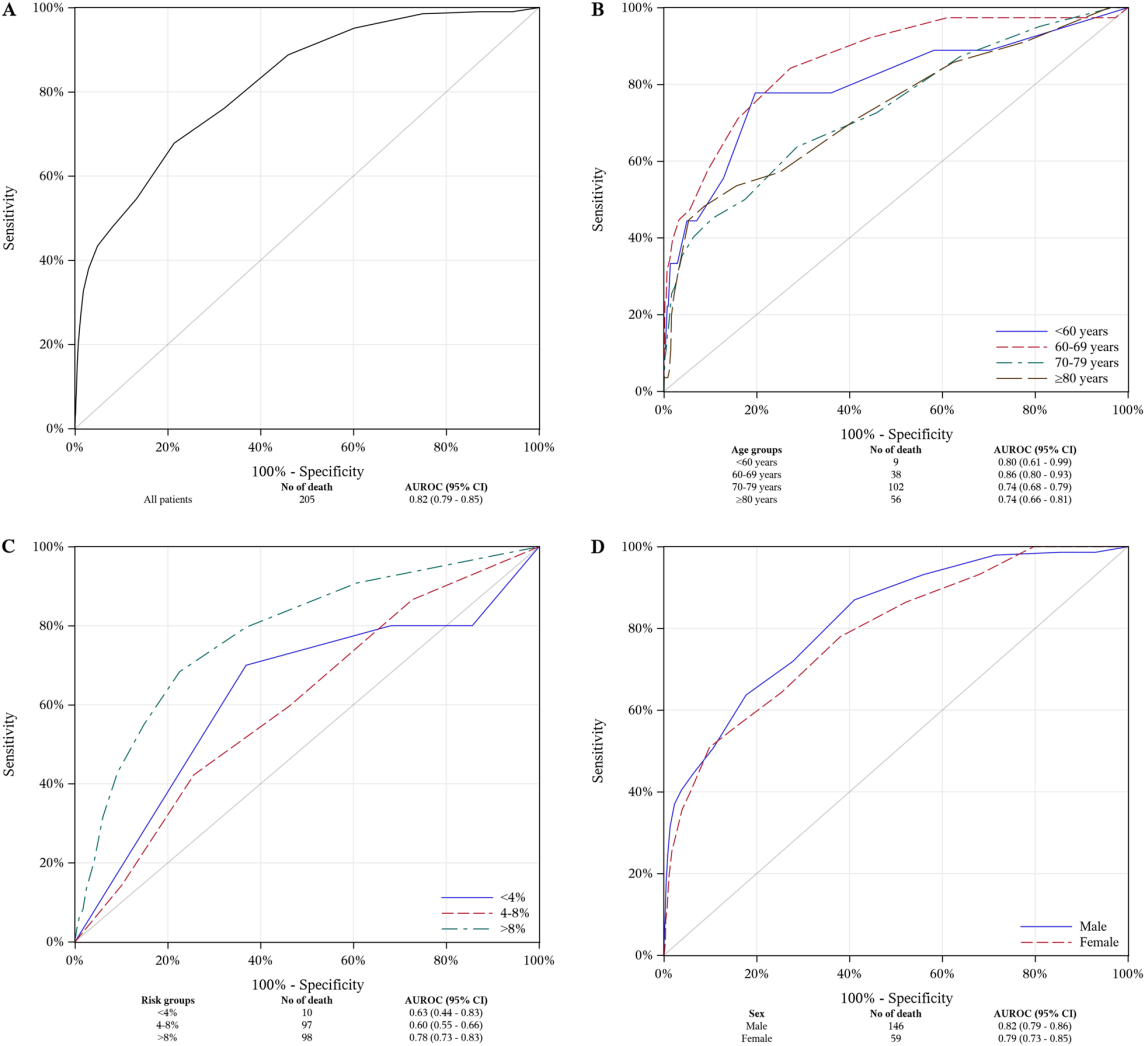
Panel **A** Average area under the receiver operating characteristic curve (AUC) for EuroSCORE II in the total group; Panel **B** AUC for EuroSCORE II by age group; Panel **C** AUC for EuroSCORE II by surgical risk; Panel **D** AUC for Euroscore II by sex

The overall discriminative accuracy of EuroSCORE II in the study population was good (AUC: 0.82; 95% CI 0.79–0.85; Fig. 1A). The accuracy of the model was acceptable in all age groups. The highest accuracy was observed in patients aged 60–69 years (AUC: 0.86, 95% CI 0.80–0.93], followed by those aged 70–79 years (AUC: 0.74, 95% CI 0.68–0.79) and > 80 years (AUC: 0.74, 95% CI 0.66–0.81; Fig. 1B).

**Fig. 2:**
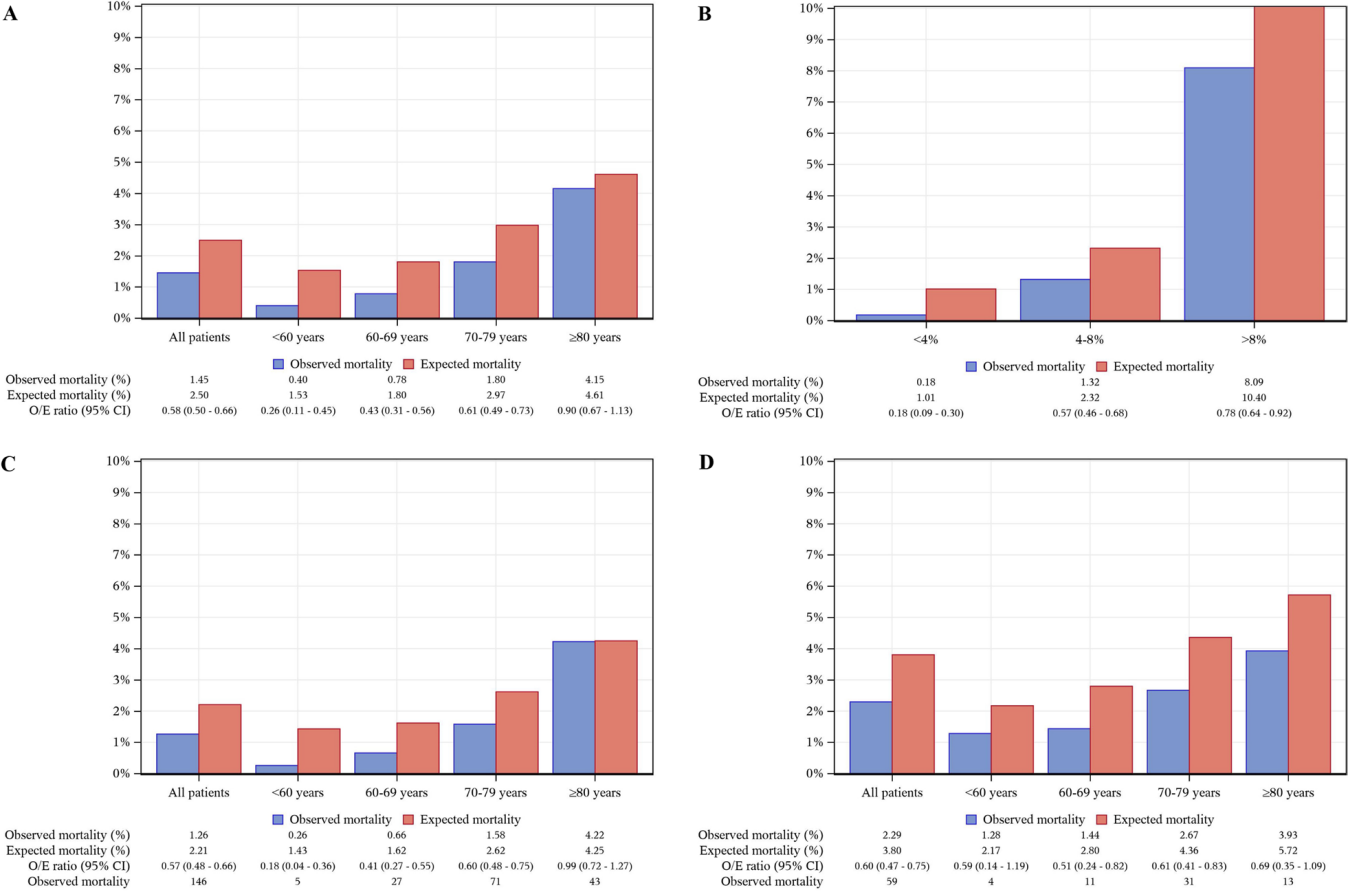
Panel **A** Observed and expected (O/E) 30-day mortality by age group in the total group; Panel **B** O/E 30-day mortality by surgical risk group; Panel **C** O/E 30-day mortality by age group among men; Panel **D** O/E 30-day mortality by age group among women

Figure 2A shows the calibration of the EuroSCORE II model in CABG patients by age. The O/E ratio for all ages was 0.58 (95% CI 0.50–0.66). The EuroSCORE II model overestimated the mortality risk in all age groups. The calibration was poorest in patients younger than 60 years, and improved with increasing age (< 60 years: O/E = 0.26 [95% CI 0.11–0.45], 60–69 years: 0.43 [0.31–0.56], 70–79 years: 0.61 [0.49–0.73], ≥ 80 years: 0.90 [0.67–1.13]).

### Performance of the EuroSCORE II model in CABG patients by sex

The discriminative accuracy of the EuroSCORE II model was acceptable in both men (AUC: 0.82, 95% CI 0.79–0.86) and women (AUC: 0.79, 95% CI 0.73–0.85) (Fig. 1D). Figures 2C and D show the calibration of the EuroSCORE II model in men versus women. The O/E ratio was 0.57 (95% CI 0.48–0.66) for men and 0.60 (95% CI 0.47–0.75) for women. Among men, the best calibration was observed in patients aged > 80 years (O/E: 0.99, 95% CI 0.72–1.27) and the lowest in patients aged < 60 years (O/E: 0.18, 95% CI 0.04–0.36). Among women, the best calibration was again observed among patients aged > 80 years (O/E: 0.69, 95% CI 0.35–1.09), while the lowest calibration was in aged 60–69 years (O/E: 0.51, 95% CI 0.24–0.82).

### Performance of the EuroSCORE II model in CABG patients by surgical risk group

The discriminative accuracy of EuroSCORE II was best among patients with high surgical risk (AUC: 0.78, 95% CI 0.73–0.83; Fig. 1C), and was lower among patients with intermediate surgical risk (AUC: 0.60, 95% CI 0.55–0.66) and low surgical risk (AUC: 0.63, 95% CI 0.44–0.83). Patients in the high-risk group had the highest O/E ratio (0.78, 95% CI 0.64–0.92), followed by patients with intermediate risk (O/E: 0.57, 95% CI 0.46–0.68) and low risk (O/E: 0.18, 95% CI 0.09–0.30) (Fig. 2B).

## Discussion

In this population-based study, we investigated the discrimination accuracy and calibration of the EuroSCORE II risk stratification tool in a large nationwide cohort of CABG patients. The main findings were as follows. Firstly, EuroSCORE II had good discriminative accuracy independently of sex and age, but markedly overestimated the mortality risk, especially in younger patients. Secondly, the discriminative accuracy and calibration were better in high-risk patients than in low-risk and intermediate-risk patients.

Risk stratification tools are used to determine the mortality risk in individual patients, but can also be used to facilitate operation program planning by optimizing patient mix, for quality assessment, and in benchmarking for comparisons between centres and surgeons. To achieve this, the tool needs to have high discriminative accuracy. The present study showed that EuroSCORE II had good discriminative accuracy when applied to a nationwide CABG cohort, and that the accuracy was mainly independent of age and sex. The overall AUC in the present study (0.82) was comparable to the accuracy achieved in the original validation data set of EuroSCORE II [3]. The acceptable overall discriminative accuracy of EuroSCORE II has been confirmed in several studies in different cardiac surgery populations as well as in meta-analyses [6, 7], showing an AUC of 0.77–0.81. The present study showed that the best discriminatory accuracy was detected in patients aged 60–69 years, and that this accuracy decreased somewhat with increasing age. These results are in accordance with those of Poullis et al., who suggested that the EuroSCORE II tool should be used with caution in patients > 70 years old [12]. The present finding of highest accuracy in patients aged 60–70 years can likely be explained by overrepresentation of patients of this age in the original EuroSCORE II dataset that was used to develop the score [3].

Besides the good discriminatory accuracy of EuroSCORE II, the results from the present study showed a marked overestimation of mortality in our CABG population, with an overall O/E ratio of 0.57. In comparison, a meta-analysis based on 22 studies in 145,592 mixed cardiac surgery patients reported an O/E ratio of 1.02 [6], while a large study in 16,096 CABG patients found an O/E ratio of 0.72 [16]. The present study does not give any clear explanation for the lower observed mortality in our study, though it may be at least partly due to improved intraoperative and postoperative care in this more contemporary study population. Nevertheless, the results imply that EuroSCORE II needs to be calibrated for different populations and/or procedures.

We observed the lowest O/E ratio in younger patients, with a value of 0.26 for patients < 60 years and 0.42 for patients aged 60–69 years, while the ratio was 0.89 in patients ≥ 80 years. This was a surprising result, given that some smaller studies have indicated that EuroSCORE II overestimates mortality in octogenarians [9, 10, 13]. Hence, our hypothesis that EuroSCORE II would perform less well in octogenarians could not be confirmed, since the discrimination accuracy was only somewhat lower in older patients and the calibration was better. We also hypothesized that EuroSCORE II would perform less well in patients with high surgical risk. This hypothesis was based on a study by Howell et al. [15] which showed low discriminative accuracy in high-risk patients (AUC: 0.65), and another study by Osnabrugge et al. [16] demonstrating a low O/E ratio (0.51) in high-risk aortic valve replacement patients. The results of the present study did not support our hypothesis, since both the discrimination accuracy and the calibration were better in high-risk than in low-risk and intermediate-risk patients.

The present study has both strengths and limitations. Strengths include the large nationwide study cohort, which is by far the largest yet used to examine the performance of EuroSCORE in relation to all three of age, sex, and surgical risk. Limitations include the definition of high, intermediate, and low surgical risk, which was adapted from the EACTS/ESC guideline definition in aortic valve replacement patients [22]. A consensus definition in CABG patients is lacking.

## Conclusion

EuroSCORE II showed a satisfactory discriminative accuracy when applied in a large cohort of CABG patients. However, it markedly overestimated the mortality risk in this study cohort, especially in younger patients. This poor calibration strongly suggests that it is necessary to calibrate EuroSCORE II for different study populations.

## Supplementary Information

**Additional file 1**. Tables S1 and S2.

## Data Availability

The data underlying this article will be shared on reasonable request to the corresponding author.

## Abbreviations

AUC: Area under the receiver operating characteristic curve
BMI: Body mass index
CABG: Coronary artery bypass grafting
CCS: Canadian cardiovascular society functional classification of angina
CI: Confidence interval
EuroSCORE: The European system for cardiac operative risk evaluation
LVEF: Left ventricular function
NYHA: New York heart association class of heart failure.
O/E: Observed/expected ratio
SWEDEHEART: Swedish web system for enhancement and development of evidence-based care in heart disease evaluated according to recommended therapies
