# Supplementary material for: The performance of EuroSCORE II in CABG patients in relation to sex, age, and surgical risk: a nationwide study in 14,118 patients
Source: J Cardiothorac Surg. 2023 Jan 19;18:40. doi: 10.1186/s13019-023-02141-4 (PMC9850511; doi:10.1186/s13019-023-02141-4)
Supplement: Supplementary file 1 — Additional file 1. Tables S1 and S2. [file 13019_2023_2141_MOESM1_ESM.docx]

SUPPLEMENTAL MATERIAL

The performance of EuroSCORE II in CABG patients in relation to sex, age, and surgical risk: a nationwide study in 14298 patients

Martin Silverborn, Susanne J Nielsen, Martin Karlsson

**Supplementary Table 1.** Baseline demographics and EuroSCORE II variables by risk group stratified by sex.

|  | **Men**  **n=11 545** | | | **Women**  **n=2569** | | |
| --- | --- | --- | --- | --- | --- | --- |
|  | **EuroSCORE II**  **<4%**  **n (%)** | **EuroSCORE II**  **4–8%**  **n (%)** | **EuroSCORE II**  **>8%**  **n (%)** | **EuroSCORE II**  **<4%**  **n (%)** | **EuroSCORE II**  **4–8%**  **n (%)** | **EuroSCORE II**  **>8%**  **n (%)** |
| No of patients | 5039 (43.6) | 5717 (49.5) | 789 (6.8) | 510 (19.9) | 1637 (63.7) | 422 (16.4) |
| Age at operation, mean (SD) | 62.7 (7.5) | 72.0 (7.4) | 74.4 (8.2) | 60.2 (7.0) | 71.8 (7.4) | 75.6 (7.1) |
| **Medical history** |  |  |  |  |  |  |
| Previous heart surgery | 3 (0.1) | 80 (1.4) | 59 (7.5) | 0 (0.0) | 11 (0.7) | 23 (5.5) |
| BMI kg/m^2^, mean (SD) | 28.1 (4.1) | 27.3 (3.8) | 26.8 (4.0) | 28.4 (5.2) | 27.7 (4.9) | 27.1 (4.8) |
| Preoperative dialysis | 41 (0.8) | 75 (1.3) | 28 (3.5) | 6 (1.2) | 29 (1.8) | 17 (4.0) |
| Previous PCI | 922 (18.3) | 1109 (19.4) | 156 (19.8) | 79 (15.5) | 237 (14.5) | 80 (19.0) |
| Recent myocardial infarction | 1027 (20.4) | 3598 (62.9) | 720 (91.3) | 72 (14.1) | 911 (55.7) | 383 (90.8) |
| Diabetes | 1297 (25.7) | 1700 (29.8) | 265 (33.7) | 176 (34.5) | 549 (33.5) | 149 (35.4) |
| Hypertension | 964 (73.4) | 1240 (78.6) | 163 (80.7) | 102 (80.3) | 353 (79.5) | 74 (80.4) |
| Atrial fibrillation | 117 (3.2) | 371 (9.0) | 89 (17.1) | 4 (1.1) | 67 (5.7) | 41 (14.9) |
| Previous stroke | 189 (3.8) | 504 (8.8) | 149 (18.9) | 22 (4.3) | 145 (8.9) | 70 (16.6) |
| Chronic respiratory disease | 174 (3.5) | 549 (9.6) | 152 (19.3) | 17 (3.3) | 192 (11.7) | 81 (19.3) |
| Extracardiac arteriopathy | 56 (1.1) | 525 (9.2) | 251 (31.9) | 5 (1.0) | 141 (8.6) | 125 (29.7) |
| Serum-creatinine, mean (SD) | 86.1 (29.1) | 96.6 (56.0) | 122.0 (105.4) | 74.7 (49.1) | 81.4 (50.0) | 93.5 (66.9) |
| Poor mobility | 19 (0.4) | 146 (2.6) | 99 (12.6) | 1 (0.2) | 28 (1.7) | 58 (13.8) |
| Critical preoperative state | 1 (0.0) | 27 (0.5) | 189 (24.0) | 0 (0.0) | 7 (0.4) | 75 (17.8) |
| NYHA class |  |  |  |  |  |  |
| I | 1447 (28.8) | 1283 (22.4) | 95 (12.1) | 127 (25.0) | 343 (21.0) | 50 (11.8) |
| II | 1933 (38.4) | 1899 (33.2) | 158 (20.1) | 203 (39.9) | 508 (31.1) | 94 (22.3) |
| III | 1418 (28.2) | 2040 (35.7) | 301 (38.2) | 155 (30.5) | 614 (37.5) | 158 (37.4) |
| IV | 121 (2.4) | 320 (5.6) | 201 (25.5) | 14 (2.8) | 120 (7.3) | 96 (22.7) |
| CCS class 4 angina | 369 (7.4) | 804 (14.2) | 310 (39.5) | 40 (7.8) | 257 (15.8) | 168 (40.2) |
| Left ventricular function |  |  |  |  |  |  |
| Normal | 4290 (85.2) | 3402 (59.5) | 171 (21.7) | 471 (92.4) | 1171 (71.5) | 120 (28.4) |
| 31%–50% | 719 (14.3) | 1978 (34.6) | 340 (43.1) | 39 (7.6) | 435 (26.6) | 212 (50.2) |
| 21%–30% | 19 (0.4) | 285 (5.0) | 223 (28.3) | 0 (0.0) | 27 (1.6) | 75 (17.8) |
| ≤20% | 10 (0.2) | 50 (0.9) | 55 (7.0) | 0 (0.0) | 4 (0.2) | 15 (3.6) |
| Pulmonary hypertension |  |  |  |  |  |  |
| <30 mmHg | 4182 (96.3) | 4559 (92.7) | 515 (78.4) | 430 (97.3) | 1275 (91.9) | 295 (83.1) |
| 30–55 mmHg | 156 (3.6) | 316 (6.4) | 110 (16.7) | 12 (2.7) | 105 (7.6) | 43 (12.1) |
| >55 mmHg | 3 (0.1) | 45 (0.9) | 32 (4.9) | 0 (0.0) | 7 (0.5) | 17 (4.8) |
| Urgency of the procedure |  |  |  |  |  |  |
| Elective | 3153 (63.0) | 2405 (42.5) | 132 (16.9) | 313 (61.5) | 711 (44.1) | 75 (18.0) |
| Urgent | 1840 (36.7) | 3076 (54.3) | 372 (47.6) | 195 (38.3) | 871 (54.0) | 218 (52.4) |
| Emergency | 14 (0.3) | 170 (3.0) | 241 (30.8) | 1 (0.2) | 30 (1.9) | 102 (24.5) |
| Salvage | 0 (0.0) | 14 (0.2) | 37 (4.7) | 0 (0.0) | 2 (0.1) | 21 (5.0) |
| ECC | 4993 (99.1) | 5641 (98.7) | 772 (97.8) | 504 (98.8) | 1611 (98.4) | 410 (97.2) |
| **Dead within 30 days** | 10 (0.2) | 71 (1.2) | 65 (8.2) | 0 (0.0) | 26 (1.6) | 33 (7.8) |
| Categorical variables presented as n (%), continuous variables presented as mean (SD) or number.  BMI = body mass index; NYHA class = New York Heart Association class of heart failure; CCS = Canadian Cardiovascular Society Functional Classification of Angina; ECC = extra corporal circulation | | | | | |  |

**Supplementary Table 2.** Baseline demographics and EuroSCORE II variables by risk group.

|  | | **EuroSCORE II**  **<4%**  **n (%)** | **EuroSCORE II**  **4–8%**  **n (%)** | **EuroSCORE II**  **>8%**  **n (%)** |
| --- | --- | --- | --- | --- |
| No of patients | | 5549 | 7354 | 1211 |
| Age at operation, mean (SD) | 62.5 (7.5) | 72.0 (7.4) | 74.9 (7.9) | |
| Sex |  |  |  | |
| Men | 5039 (90.8) | 5717 (77.7) | 789 (65.2) | |
| Women | 510 (9.2) | 1637 (22.3) | 422 (34.8) | |
| **Medical history** |  |  |  | |
| Previous heart surgery | 3 (0.1) | 91 (1.2) | 82 (6.8) | |
| BMI in kg/m^2^, mean (SD) | 28.2 (4.2) | 27.4 (4.1) | 26.9 (4.3) | |
| Preoperative dialysis | 47 (0.8) | 104 (1.4) | 45 (3.7) | |
| Previous PCI | 1001 (18.1) | 1346 (18.3) | 236 (19.5) | |
| Recent myocardial infarction | 1099 (19.8) | 4509 (61.3) | 1103 (91.1) | |
| Diabetes | 1473 (26.6) | 2249 (30.6) | 414 (34.3) | |
| Hypertension | 1066 (74.0) | 1593 (78.8) | 237 (80.6) | |
| Atrial fibrillation | 121 (3.0) | 438 (8.2) | 130 (16.3) | |
| Previous stroke | 211 (3.8) | 649 (8.8) | 219 (18.1) | |
| Chronic respiratory disease | 191 (3.4) | 741 (10.1) | 233 (19.3) | |
| Extracardiac arteriopathy | 61 (1.1) | 666 (9.1) | 376 (31.1) | |
| Serum-creatinine, mean (SD) | 85.1 (31.7) | 93.2 (55.1) | 112.1 (94.7) | |
| Poor mobility | 20 (0.4) | 174 (2.4) | 157 (13.0) | |
| Critical preoperative state | 1 (0.0) | 34 (0.5) | 264 (21.8) | |
| NYHA class |  |  |  | |
| I | 1574 (28.4) | 1626 (22.1) | 145 (12.0) | |
| II | 2136 (38.5) | 2407 (32.7) | 252 (20.8) | |
| III | 1573 (28.4) | 2654 (36.1) | 459 (38.0) | |
| IV | 135 (2.4) | 440 (6.0) | 297 (24.6) | |
| CCS class 4 angina | 409 (7.4) | 1061 (14.5) | 478 (39.8) | |
| Left ventricular function |  |  |  | |
| Normal | 4761 (85.8) | 4573 (62.2) | 291 (24.0) | |
| 31%–50% | 758 (13.7) | 2413 (32.8) | 552 (45.6) | |
| 21%–30% | 19 (0.3) | 312 (4.2) | 298 (24.6) | |
| <20% | 10 (0.2) | 54 (0.7) | 70 (5.8) | |
| Pulmonary hypertension |  |  |  | |
| <30 mm Hg | 4612 (96.4) | 5834 (92.5) | 810 (80.0) | |
| 30–55 mm Hg | 168 (3.5) | 421 (6.7) | 153 (15.1) | |
| >55 mm Hg | 3 (0.1) | 52 (0.8) | 49 (4.8) | |
| Urgency of the procedure |  |  |  | |
| Elective | 3466 (62.8) | 3116 (42.8) | 207 (17.3) | |
| Urgent | 2035 (36.9) | 3947 (54.2) | 590 (49.2) | |
| Emergency | 15 (0.3) | 200 (2.7) | 343 (28.6) | |
| Salvage | 0 (0.0) | 16 (0.2) | 58 (4.8) | |
| ECC | 5497 (99.1) | 7252 (98.6) | 1182 (97.6) | |
| Dead within 30 days |  |  |  | |
| All patients | 10 (0.2) | 97 (1.3) | 98 (8.0) | |
| Men | 10 (0.2) | 71 (1.2) | 65 (8.2) | |
| Women | | 0 (0.0) | 26 (1.6) | 33 (7.8) |
| Categorical variables presented as n (%), continuous variables presented as mean (SD) or number. BMI = body mass index; NYHA class = New York Heart Association class of heart failure; CCS = Canadian Cardiovascular Society Functional Classification of Angina; ECC = extra corporal circulation. | | | | |
